# Prize Question in Germany

**Published:** 1861-07

**Authors:** 


PRIZE QUESTION IN GERMANY.—The German Society of Psychiatrie and Juridical Psychology offers a prize of one hundred thalers for the best essay on the following subject: "What is the Classification of Mental Disturbances which is likely to prove most Serviceable in regard to Practical Medicine?" Essays to be sent to Dr. Erlenmeyer, secretary to the society, at Bendorf, near Coblenz, in the course of the present year.

